# Supplementary material for: Modification of High-Density Lipoprotein Functions by Diet and Other Lifestyle Changes: A Systematic Review of Randomized Controlled Trials
Source: J Clin Med. 2021 Dec 15;10(24):5897. doi: 10.3390/jcm10245897 (PMC8707678; doi:10.3390/jcm10245897)
Supplement: Supplementary file 1 [file jcm-10-05897-s001.zip › jcm-1495039-supplementary.pdf]

ON-LINE SUPPLEMENTARY MATERIAL

Table S1. Risk of bias evaluation

Supplementary File S1. List of terms used for article selection

Table S1. Risk of bias evaluation

First author (year)	Random sequence generation	Allocation concealment	Blinding of participants and personnel	Blinding of outcome assessment	Incomplete outcome data	Selective reporting	Other potential bias	Overall risk of bias
Thomas TR (1985)	?	?	?	?	?	?	?	?
Baudet MF (1988)	?	?	?	?	?	?	?	-
Rönnemaa T (1988)	?	?	?	?	?	?	?	?
Abbey M (1990)	?	?	?	?	+	+	?	?
Singer P (1990)	?	?	?	?	?	?	?	?
Williams PT (1990)	?	+	-	?	+	+	+	+
Vorster HH (1992)	-	?	-	+	?	+	+	-
Martin LJ (1993)	?	?	-	?	?	+	+	?
Cox C (1994)	?	?	-	?	+	?	+	?
Ginsberg HN (1994)	?	?	+	+	-	+	+	-

First author (year)	Random sequence generation	Allocation concealment	Blinding of participants and personnel	Blinding of outcome assessment	Incomplete outcome data	Selective reporting	Other potential bias	Overall risk of bias
Cox C (1995)	?	?	-	?	+	+	+	?
Ginsberg HN (1995)	?	?	+	+	-	+	+	-
Schwab US (1995)	?	?	-	?	?	+	-	-
van Tol A (1995)	-	?	-	?	?	+	?	-
Lottenberg AM (1996)	?	?	+	-	?	+	+	?
Solà R (1997)	?	?	-	?	?	+	+	?
Blanco-Molina A (1998)	?	?	-	+	+	+	+	?
Miida T (1998)	?	?	-	?	?	+	+	-
Lagrost L (1999)	?	?	+	?	-	+	+	?
Pownall HJ (1999)	?	?	+	?	?	+	+	?
Van der Gaag MS (1999)	?	?	-	?	-	+	?	?

First author (year)	Random sequence generation	Allocation concealment	Blinding of participants and personnel	Blinding of outcome assessment	Incomplete outcome data	Selective reporting	Other potential bias	Overall risk of bias
Senault C (2000)	?	?	?	?	?	?	?	?
Higashi K (2001)	?	?	-	?	-	-	?	?
Lichtenstein AH (2001)	?	?	+	+	?	+	+	?
Matthan NR (2001)	?	?	+	+	-	+	+	?
Van der Gaag MS (2001)	?	?	-	?	-	+	?	?
Vega-López S (2001)	?	?	?	?	+	+	+	?
Bub A (2002)	?	?	-	?	+	+	?	?
de Roos NM (2002)	?	?	-	+	+	+	-	-
Freese R (2002)	?	?	+	+	+	+	+	+
Herron KL (2002)	?	?	-	?	?	+	+	?
Lichtenstein AH (2002)	?	?	+	+	?	+	+	?

First author (year)	Random sequence generation	Allocation concealment	Blinding of participants and personnel	Blinding of outcome assessment	Incomplete outcome data	Selective reporting	Other potential bias	Overall risk of bias
Rantala M (2002)	?	?	-	?	?	+	+	?
Sierksma A (2002)	?	?	-	-	+	+	+	-
Herron KL (2003)	?	?	-	?	?	+	+	?
Homma Y (2003)	?	+	+	+	?	+	+	?
Lottenberg AM (2003)	?	?	+	?	?	+	-	-
Beulens JW (2004)	?	?	-	-	+	+	?	?
Calabresi (2004)	?	?	+	?	?	+	?	?
Chung BH (2004)	?	?	-	?	?	+	+	?
Herron KL (2004)	?	?	-	?	?	+	+	?
Sierksma A (2004)	?	?	-	-	+	+	+	-
Tholstrup T (2004)	?	?	+	?	+	+	+	?

First author (year)	Random sequence generation	Allocation concealment	Blinding of participants and personnel	Blinding of outcome assessment	Incomplete outcome data	Selective reporting	Other potential bias	Overall risk of bias
Bub A (2005)	?	?	-	?	+	+	?	?
Cherki M (2005)	?	?	-	?	+	+	+	?
Zern TL (2005)	?	?	+	?	?	+	+	?
Suomela JP (2006)	?	?	+	?	-	+	+	?
Vega-López S (2006)	?	?	?	?	?	+	+	?
Wood RJ (2006)	?	?	+	?	+	+	+	+
Buonacorso V (2007)	?	?	-	?	?	+	?	?
Canales A (2007)	?	?	-	?	?	+	+	?
Dalgård C (2007)	?	?	-	+	-	+	-	-
Shrestha S (2007)	?	?	+	?	+	+	+	+
Waters D (2007)	?	?	?	?	?	-	+	?

First author (year)	Random sequence generation	Allocation concealment	Blinding of participants and personnel	Blinding of outcome assessment	Incomplete outcome data	Selective reporting	Other potential bias	Overall risk of bias
Chardigny JM (2008)	+	?	+	?	-	+	+	+
Gebauer SK (2008)	?	?	+	+	+	+	+	+
Kralova-Lesna I (2008)	?	?	-	?	?	+	+	-
Ozdemir B (2008)	?	?	-	?	?	+	-	-
Puglisi MJ (2009)	+	?	-	?	?	+	+	?
Qin Y (2009)	?	?	+	?	+	+	+	+
Shidfar F (2009)	+	?	+	?	?	+	+	?
Vega-López S (2009)	?	?	-	?	-	+	+	?
Vislocky LM (2009)	?	?	-	?	+	+	+	?
Králová Lesná I (2010)	?	?	-	?	?	+	-	-
Mutungi G (2010)	?	?	+	-	+	+	+	?

First author (year)	Random sequence generation	Allocation concealment	Blinding of participants and personnel	Blinding of outcome assessment	Incomplete outcome data	Selective reporting	Other potential bias	Overall risk of bias
Canales A (2011)	?	?	-	?	-	+	+	?
Pfeuffer M (2011)	?	?	+	?	?	+	+	?
Ghorbanihaghjo A (2012)	+	?	+	?	+	+	+	+
Qian Q (2012)	?	+	+	?	-	+	+	+
Sánchez-Muniz FJ (2012)	?	?	-	?	?	+	+	?
Andersen CJ (2013)	?	?	+	?	?	+	?	?
Baralic I (2013)	?	?	+	?	?	+	?	?
Blesso CN (2013)	?	?	+	?	?	-	?	?
Damasceno NR (2013)	+	+	-	+	?	+	+	+
McEneny J (2013)	+	?	+	-	+	+	+	+
Zhu Y (2013)	?	+	+	?	+	+	+	+

First author (year)	Random sequence generation	Allocation concealment	Blinding of participants and personnel	Blinding of outcome assessment	Incomplete outcome data	Selective reporting	Other potential bias	Overall risk of bias
Daniels JA (2014)	?	?	-	+	-	+	?	?
Hernáez A (2014)	+	?	-	+	+	+	+	+
Holligan SD (2014)	?	?	-	+	+	+	+	+
Stirban A (2014)	?	?	+	?	?	+	+	?
Farràs M (2015)	+	?	+	+	+	+	+	+
Kawakami Y (2015)	?	?	+	?	-	+	+	?
Shidfar F (2015)	+	?	+	+	+	+	-	+
Shidfar F (2015)	+	?	+	+	+	+	-	+
Wu PT (2015)	?	?	+	+	-	+	+	+
Tiainen S (2016)	+	+	-	+	+	+	+	+
Wesnigk J (2016)	-	?	-	?	-	+	?	-

First author (year)	Random sequence generation	Allocation concealment	Blinding of participants and personnel	Blinding of outcome assessment	Incomplete outcome data	Selective reporting	Other potential bias	Overall risk of bias
Albaghdadi MS (2017)	+	?	-	+	+	+	+	+
Berryman CE (2017)	+	?	-	-	-	+	?	-
Dokras A (2017)	+	+	-	-	+	+	-	?
Fernández-Castillejo S (2017)	+	?	+	+	+	+	+	+
Golzari MH (2017)	+	?	+	?	?	+	?	?
Hernaez A (2017)	+	+	-	+	+	+	+	+
Lambert C (2017)	?	?	+	?	?	+	+	?
Brassard (2018)	?	?	+	+	-	+	+	?
de Souza (2018)	-	+	-	+	-	+	+	-
Farràs M (2018)	+	?	+	+	+	+	+	+
Khan AA (2018)	?	?	-	?	?	+	+	?

First author (year)	Random sequence generation	Allocation concealment	Blinding of participants and personnel	Blinding of outcome assessment	Incomplete outcome data	Selective reporting	Other potential bias	Overall risk of bias
Lazavi F (2018)	+	+	-	?	+	+	+	+
Liu X (2018)	+	+	+	?	-	+	+	+
Meng (2018)	?	?	-	+	?	+	+	?
Millar (2018)	?	?	+	+	?	?	+	?
Missimer (2018)	?	?	-	+	+	+	+	?
Morgantini (2018)	+	?	-	-	+	+	+	+
Padro T (2018)	?	?	-	-	+	+	+	?
Richter CK (2018)	?	+	+	+	+	+	+	+
Sarzynski (2018)	+	+	-	-	-	+	+	-
Talbot (2018)	?	?	-	?	+	+	+	?
Woudberg NJ (2018)	+	+	-	?	-	+	+	+

First author (year)	Random sequence generation	Allocation concealment	Blinding of participants and personnel	Blinding of outcome assessment	Incomplete outcome data	Selective reporting	Other potential bias	Overall risk of bias
Wurm R (2018)	+	+	+	?	+	+	+	+
Balsan (2019)	+	+	-	+	?	+	+	+
Manninen (2019)	+	?	-	-	+	+	-	-
Michaličková (2019)	?	?	+	?	?	+	+	?
Sawrey-Kubicek (2019)	+	?	+	-	+	+	+	+
Andraski (2020)	?	?	-	?	+	+	+	?
Favari E (2020)	+	?	+	?	+	+	+	+
Hernaez A (2020)	+	+	-	+	+	+	+	+
Tindall AM (2020)	+	?	-	?	-	+	+	-

(+): Low risk of bias. (-): High risk of bias. (?): Unclear risk of bias.

Supplementary File S1. List of terms used for article selection

1. Cholesterol efflux capacity

(high density [Title/Abstract] OR high-density [Title/Abstract] OR HDL [Title/Abstract] OR lipoprotein* [Title/Abstract]) AND cholesterol efflux [Title/Abstract]) AND (diet* [Title/Abstract] OR nutr* [Title/Abstract] OR fatty acid* [Title/Abstract] OR oil [Title/Abstract] OR *saturated [Title/Abstract] OR trans [Title/Abstract] OR MUFA* [Title/Abstract] OR PUFA* [Title/Abstract] OR omega* [Title/Abstract] OR antioxidant [Title/Abstract] OR vitamin [Title/Abstract] OR tocopher* [Title/Abstract] OR ascorb* [Title/Abstract] OR lycopene [Title/Abstract] OR *phenol [Title/Abstract] OR *phenols [Title/Abstract] OR phenol* [Title/Abstract] OR flavonoid* [Title/Abstract] OR fruit* [Title/Abstract] OR vegeta* [Title/Abstract] OR juice* [Title/Abstract] OR *berry [Title/Abstract] OR olive [Title/Abstract] OR tomato [Title/Abstract] OR tea [Title/Abstract] OR coffee [Title/Abstract] OR whole grain* [Title/Abstract] OR legume* [Title/Abstract] OR fish [Title/Abstract] OR egg* [Title/Abstract] OR nut [Title/Abstract] OR walnut* [Title/Abstract] OR *nuts [Title/Abstract] OR low fat [Title/Abstract] OR protein-rich [Title/Abstract] OR Mediterranean diet [Title/Abstract] OR ethanol* [Title/Abstract] OR alcohol* [Title/Abstract] OR beer [Title/Abstract] OR wine [Title/Abstract] OR lifestyle [Title/Abstract] OR physical activity [Title/Abstract] OR exercise [Title/Abstract] OR training [Title/Abstract] OR weight loss [Title/Abstract] OR restrict* [Title/Abstract] OR very low calorie [Title/Abstract] OR Eicosapentaenoic [Title/Abstract] OR EPA [Title/Abstract] OR Docosahexaenoic [Title/Abstract] OR DHA [Title/Abstract] OR Flavonol* [Title/Abstract] OR Lignan* [Title/Abstract] OR Oleic [Title/Abstract] OR soy* [Title/Abstract] OR catechin* [Title/Abstract] OR DASH diet* [Title/Abstract] OR plant sterol* [Title/Abstract] OR grape* [Title/Abstract] OR Probiotic [Title/Abstract] OR Pistachio [Title/Abstract] OR Resveratrol [Title/Abstract] OR Milk [Title/Abstract] OR Beef [Title/Abstract] OR Fiber* [Title/Abstract] OR inulin [Title/Abstract] OR margarine* [Title/Abstract] OR Garlic [Title/Abstract]) AND humans [MeSH Terms]

2. CETP activity or mass

(CETP [Title/Abstract] OR "cholesteryl ester*"[Title/Abstract] OR "cholesterol ester"[Title/Abstract]) AND (diet* [Title/Abstract] OR nutr* [Title/Abstract] OR fatty acid* [Title/Abstract] OR oil [Title/Abstract] OR *saturated [Title/Abstract] OR trans [Title/Abstract] OR MUFA* [Title/Abstract] OR PUFA* [Title/Abstract] OR omega* [Title/Abstract] OR antioxidant [Title/Abstract] OR vitamin [Title/Abstract] OR tocopher* [Title/Abstract] OR ascorb* [Title/Abstract] OR lycopene [Title/Abstract] OR *phenol [Title/Abstract] OR *phenols [Title/Abstract] OR phenol* [Title/Abstract] OR flavonoid*

[Title/Abstract] OR fruit* [Title/Abstract] OR vegeta* [Title/Abstract] OR juice* [Title/Abstract] OR *berry [Title/Abstract] OR olive [Title/Abstract] OR tomato [Title/Abstract] OR tea [Title/Abstract] OR coffee [Title/Abstract] OR whole grain* [Title/Abstract] OR legume* [Title/Abstract] OR fish [Title/Abstract] OR egg* [Title/Abstract] OR nut [Title/Abstract] OR walnut* [Title/Abstract] OR *nuts [Title/Abstract] OR low fat [Title/Abstract] OR protein-rich [Title/Abstract] OR Mediterranean diet [Title/Abstract] OR ethanol* [Title/Abstract] OR alcohol* [Title/Abstract] OR beer [Title/Abstract] OR wine [Title/Abstract] OR lifestyle [Title/Abstract] OR physical activity [Title/Abstract] OR exercise [Title/Abstract] OR training [Title/Abstract] OR weight loss [Title/Abstract] OR restrict* [Title/Abstract] OR very low calorie [Title/Abstract] OR Eicosapentaenoic [Title/Abstract] OR EPA [Title/Abstract] OR Docosahexaenoic [Title/Abstract] OR DHA [Title/Abstract] OR Flavonol* [Title/Abstract] OR Lignan* [Title/Abstract] OR Oleic [Title/Abstract] OR soy* [Title/Abstract] OR catechin* [Title/Abstract] OR DASH diet* [Title/Abstract] OR plant sterol* [Title/Abstract] OR grape* [Title/Abstract] OR Probiotic [Title/Abstract] OR Pistachio [Title/Abstract] OR Resveratrol [Title/Abstract] OR Milk [Title/Abstract] OR Beef [Title/Abstract] OR Fiber* [Title/Abstract] OR inulin [Title/Abstract] OR margarine* [Title/Abstract] OR Garlic [Title/Abstract]) AND humans [MeSH Terms]

3. LCAT activity or mass

(*LCAT* [Title/Abstract] OR *acyltransferase [Title/Abstract] OR *acyl-transferase [Title/Abstract] OR *acyl transferase [Title/Abstract] OR lecithin:cholesterol* [Title/Abstract] OR lecithin-cholesterol* [Title/Abstract]) AND (diet* [Title/Abstract] OR nutr* [Title/Abstract] OR fatty acid* [Title/Abstract] OR oil [Title/Abstract] OR *saturated [Title/Abstract] OR trans [Title/Abstract] OR MUFA* [Title/Abstract] OR PUFA* [Title/Abstract] OR omega* [Title/Abstract] OR antioxidant [Title/Abstract] OR vitamin [Title/Abstract] OR tocopher* [Title/Abstract] OR ascorb* [Title/Abstract] OR lycopene [Title/Abstract] OR *phenol [Title/Abstract] OR *phenols [Title/Abstract] OR phenol* [Title/Abstract] OR flavonoid* [Title/Abstract] OR fruit* [Title/Abstract] OR vegeta* [Title/Abstract] OR juice* [Title/Abstract] OR *berry [Title/Abstract] OR olive [Title/Abstract] OR tomato [Title/Abstract] OR tea [Title/Abstract] OR coffee [Title/Abstract] OR whole grain* [Title/Abstract] OR legume* [Title/Abstract] OR fish [Title/Abstract] OR egg* [Title/Abstract] OR nut [Title/Abstract] OR walnut* [Title/Abstract] OR *nuts [Title/Abstract] OR low fat [Title/Abstract] OR protein-rich [Title/Abstract] OR Mediterranean diet [Title/Abstract] OR ethanol* [Title/Abstract] OR alcohol* [Title/Abstract] OR beer [Title/Abstract] OR wine [Title/Abstract] OR lifestyle [Title/Abstract] OR physical activity [Title/Abstract] OR exercise [Title/Abstract] OR

training [Title/Abstract] OR weight loss [Title/Abstract] OR restrict* [Title/Abstract] OR very low calorie [Title/Abstract] OR Eicosapentaenoic [Title/Abstract] OR EPA [Title/Abstract] OR Docosahexaenoic [Title/Abstract] OR DHA [Title/Abstract] OR Flavonol* [Title/Abstract] OR Lignan* [Title/Abstract] OR Oleic [Title/Abstract] OR soy* [Title/Abstract] OR catechin* [Title/Abstract] OR DASH diet* [Title/Abstract] OR plant sterol* [Title/Abstract] OR grape* [Title/Abstract] OR Probiotic [Title/Abstract] OR Pistachio [Title/Abstract] OR Resveratrol [Title/Abstract] OR Milk [Title/Abstract] OR Beef [Title/Abstract] OR Fiber* [Title/Abstract] OR inulin [Title/Abstract] OR margarine* [Title/Abstract] OR Garlic [Title/Abstract]) AND humans [MeSH Terms]

4. HDL antioxidant capacity

(high density [Title] OR high-density [Title] OR HDL* [Title]) AND (*oxida* [Title/Abstract] OR redox [Title/Abstract] OR diene* [Title/Abstract] OR TBARS [Title/Abstract] OR lag time [Title/Abstract] OR lag-time [Title/Abstract] OR oxidation rate [Title/Abstract] OR HOI [Title/Abstract] OR dihydrorhodamine [Title/Abstract] OR DHR [Title/Abstract] OR DOR [Title/Abstract] OR ORAC [Title/Abstract] OR oxygen radical absorbance capacity [Title/Abstract] OR inflammatory index [Title/Abstract] OR HII [Title/Abstract]) AND (diet* [Title/Abstract] OR nutr* [Title/Abstract] OR fatty acid* [Title/Abstract] OR oil [Title/Abstract] OR *saturated [Title/Abstract] OR trans [Title/Abstract] OR MUFA* [Title/Abstract] OR PUFA* [Title/Abstract] OR omega* [Title/Abstract] OR antioxidant [Title/Abstract] OR vitamin [Title/Abstract] OR tocopher* [Title/Abstract] OR ascorb* [Title/Abstract] OR lycopene [Title/Abstract] OR *phenol [Title/Abstract] OR *phenols [Title/Abstract] OR phenol* [Title/Abstract] OR flavonoid* [Title/Abstract] OR fruit* [Title/Abstract] OR vegeta* [Title/Abstract] OR juice* [Title/Abstract] OR *berry [Title/Abstract] OR olive [Title/Abstract] OR tomato [Title/Abstract] OR tea [Title/Abstract] OR coffee [Title/Abstract] OR whole grain* [Title/Abstract] OR legume* [Title/Abstract] OR fish [Title/Abstract] OR egg* [Title/Abstract] OR nut [Title/Abstract] OR walnut* [Title/Abstract] OR *nuts [Title/Abstract] OR low fat [Title/Abstract] OR protein-rich [Title/Abstract] OR Mediterranean diet [Title/Abstract] OR ethanol* [Title/Abstract] OR alcohol* [Title/Abstract] OR beer [Title/Abstract] OR wine [Title/Abstract] OR lifestyle [Title/Abstract] OR physical activity [Title/Abstract] OR exercise [Title/Abstract] OR training [Title/Abstract] OR weight loss [Title/Abstract] OR restrict* [Title/Abstract] OR very low calorie [Title/Abstract] OR Eicosapentaenoic [Title/Abstract] OR EPA [Title/Abstract] OR Docosahexaenoic [Title/Abstract] OR DHA [Title/Abstract] OR Flavonol* [Title/Abstract] OR Lignan* [Title/Abstract] OR Oleic [Title/Abstract] OR soy* [Title/Abstract] OR catechin* [Title/Abstract] OR DASH diet* [Title/Abstract] OR plant

sterol* [Title/Abstract] OR grape* [Title/Abstract] OR Probiotic [Title/Abstract] OR Pistachio [Title/Abstract] OR Resveratrol [Title/Abstract] OR Milk [Title/Abstract] OR Beef [Title/Abstract] OR Fiber* [Title/Abstract] OR inulin [Title/Abstract] OR margarine* [Title/Abstract] OR Garlic [Title/Abstract]) AND humans [MeSH Terms]

5. HDL oxidation status

(high density [Title] OR high-density [Title] OR HDL* [Title]) AND (*oxida* [Title/Abstract] OR redox [Title/Abstract] OR diene* [Title/Abstract] OR thiobarbituric [Title/Abstract] OR TBARS [Title/Abstract] OR lag time [Title/Abstract] OR lag-time [Title/Abstract] OR oxidation rate [Title/Abstract] OR HOI [Title/Abstract] OR dihydrorhodamine [Title/Abstract] OR ORAC [Title/Abstract] OR oxygen radical absorbance capacity [Title/Abstract] OR phenol* [Title/Abstract] OR polyphenol* [Title/Abstract] OR vitamin E [Title/Abstract] OR tocopher* [Title/Abstract]) AND (diet* [Title/Abstract] OR nutr* [Title/Abstract] OR fatty acid* [Title/Abstract] OR oil [Title/Abstract] OR *saturated [Title/Abstract] OR trans [Title/Abstract] OR MUFA* [Title/Abstract] OR PUFA* [Title/Abstract] OR omega* [Title/Abstract] OR antioxidant [Title/Abstract] OR vitamin [Title/Abstract] OR tocopher* [Title/Abstract] OR ascorb* [Title/Abstract] OR lycopene [Title/Abstract] OR *phenol [Title/Abstract] OR *phenols [Title/Abstract] OR phenol* [Title/Abstract] OR flavonoid* [Title/Abstract] OR fruit* [Title/Abstract] OR vegeta* [Title/Abstract] OR juice* [Title/Abstract] OR *berry [Title/Abstract] OR olive [Title/Abstract] OR tomato [Title/Abstract] OR tea [Title/Abstract] OR coffee [Title/Abstract] OR whole grain* [Title/Abstract] OR legume* [Title/Abstract] OR fish [Title/Abstract] OR egg* [Title/Abstract] OR nut [Title/Abstract] OR walnut* [Title/Abstract] OR *nuts [Title/Abstract] OR low fat [Title/Abstract] OR protein-rich [Title/Abstract] OR Mediterranean diet [Title/Abstract] OR ethanol* [Title/Abstract] OR alcohol* [Title/Abstract] OR beer [Title/Abstract] OR wine [Title/Abstract] OR lifestyle [Title/Abstract] OR physical activity [Title/Abstract] OR exercise [Title/Abstract] OR training [Title/Abstract] OR weight loss [Title/Abstract] OR restrict* [Title/Abstract] OR very low calorie [Title/Abstract] OR Eicosapentaenoic [Title/Abstract] OR EPA [Title/Abstract] OR Docosahexaenoic [Title/Abstract] OR DHA [Title/Abstract] OR Flavonol* [Title/Abstract] OR Lignan* [Title/Abstract] OR Oleic [Title/Abstract] OR soy* [Title/Abstract] OR catechin* [Title/Abstract] OR DASH diet* [Title/Abstract] OR plant sterol* [Title/Abstract] OR grape* [Title/Abstract] OR Probiotic [Title/Abstract] OR Pistachio [Title/Abstract] OR Resveratrol [Title/Abstract] OR Milk [Title/Abstract] OR Beef [Title/Abstract] OR Fiber* [Title/Abstract] OR inulin [Title/Abstract] OR margarine* [Title/Abstract] OR Garlic [Title/Abstract]) AND humans [MeSH Terms]

6. PON1 activity and mass

(*paraoxonase* [Title/Abstract] OR PON [Title/Abstract] OR PON-1 [Title/Abstract] OR PON1 [Title/Abstract]) AND (diet* [Title/Abstract] OR nutr* [Title/Abstract] OR fatty acid* [Title/Abstract] OR oil [Title/Abstract] OR *saturated [Title/Abstract] OR trans [Title/Abstract] OR MUFA* [Title/Abstract] OR PUFA* [Title/Abstract] OR omega* [Title/Abstract] OR antioxidant [Title/Abstract] OR vitamin [Title/Abstract] OR tocopher* [Title/Abstract] OR ascorb* [Title/Abstract] OR lycopene [Title/Abstract] OR *phenol [Title/Abstract] OR *phenols [Title/Abstract] OR phenol* [Title/Abstract] OR flavonoid* [Title/Abstract] OR fruit* [Title/Abstract] OR vegeta* [Title/Abstract] OR juice* [Title/Abstract] OR *berry [Title/Abstract] OR olive [Title/Abstract] OR tomato [Title/Abstract] OR tea [Title/Abstract] OR coffee [Title/Abstract] OR whole grain* [Title/Abstract] OR legume* [Title/Abstract] OR fish [Title/Abstract] OR egg* [Title/Abstract] OR nut [Title/Abstract] OR walnut* [Title/Abstract] OR *nuts [Title/Abstract] OR low fat [Title/Abstract] OR protein-rich [Title/Abstract] OR Mediterranean diet [Title/Abstract] OR ethanol* [Title/Abstract] OR alcohol* [Title/Abstract] OR beer [Title/Abstract] OR wine [Title/Abstract] OR lifestyle [Title/Abstract] OR physical activity [Title/Abstract] OR exercise [Title/Abstract] OR training [Title/Abstract] OR weight loss [Title/Abstract] OR restrict* [Title/Abstract] OR very low calorie [Title/Abstract] OR Eicosapentaenoic [Title/Abstract] OR EPA [Title/Abstract] OR Docosahexaenoic [Title/Abstract] OR DHA [Title/Abstract] OR Flavonol* [Title/Abstract] OR Lignan* [Title/Abstract] OR Oleic [Title/Abstract] OR soy* [Title/Abstract] OR catechin* [Title/Abstract] OR DASH diet* [Title/Abstract] OR plant sterol* [Title/Abstract] OR grape* [Title/Abstract] OR Probiotic [Title/Abstract] OR Pistachio [Title/Abstract] OR Resveratrol [Title/Abstract] OR Milk [Title/Abstract] OR Beef [Title/Abstract] OR Fiber* [Title/Abstract] OR inulin [Title/Abstract] OR margarine* [Title/Abstract] OR Garlic [Title/Abstract]) AND humans [MeSH Terms]

7. HDL anti-inflammatory and endothelial protection properties

(high density [Title] OR high-density [Title] OR HDL* [Title]) AND (*inflam* [Title/Abstract] OR adhesion molecule* [Title/Abstract] OR VCAM-1 [Title/Abstract] OR VCAM1 [Title/Abstract] OR ICAM-1 [Title/Abstract] OR ICAM1 [Title/Abstract] OR monocyte chemotactic protein [Title/Abstract] OR MCP-1 [Title/Abstract] OR MCP1 [Title/Abstract] OR interleukin [Title/Abstract] OR eNOS [Title/Abstract] OR endothelial nitric oxide [Title/Abstract] OR nitric oxide[Title/Abstract] OR vasodilation [Title/Abstract] OR vascular-protective [Text Word] OR vasculoprotective

[Title/Abstract] OR vasculo-protective [Title/Abstract] OR vascular protection [Title/Abstract] OR endothelial cell [Title/Abstract]) AND (diet* [Title/Abstract] OR nutr* [Title/Abstract] OR fatty acid* [Title/Abstract] OR oil [Title/Abstract] OR *saturated [Title/Abstract] OR trans [Title/Abstract] OR MUFA* [Title/Abstract] OR PUFA* [Title/Abstract] OR omega* [Title/Abstract] OR antioxidant [Title/Abstract] OR vitamin [Title/Abstract] OR tocopher* [Title/Abstract] OR ascorb* [Title/Abstract] OR lycopene [Title/Abstract] OR *phenol [Title/Abstract] OR *phenols [Title/Abstract] OR phenol* [Title/Abstract] OR flavonoid* [Title/Abstract] OR fruit* [Title/Abstract] OR vegeta* [Title/Abstract] OR juice* [Title/Abstract] OR *berry [Title/Abstract] OR olive [Title/Abstract] OR tomato [Title/Abstract] OR tea [Title/Abstract] OR coffee [Title/Abstract] OR whole grain* [Title/Abstract] OR legume* [Title/Abstract] OR fish [Title/Abstract] OR egg* [Title/Abstract] OR nut [Title/Abstract] OR walnut* [Title/Abstract] OR *nuts [Title/Abstract] OR low fat [Title/Abstract] OR protein-rich [Title/Abstract] OR Mediterranean diet [Title/Abstract] OR ethanol* [Title/Abstract] OR alcohol* [Title/Abstract] OR beer [Title/Abstract] OR wine [Title/Abstract] OR lifestyle [Title/Abstract] OR physical activity [Title/Abstract] OR exercise [Title/Abstract] OR training [Title/Abstract] OR weight loss [Title/Abstract] OR restrict* [Title/Abstract] OR very low calorie [Title/Abstract] OR Eicosapentaenoic [Title/Abstract] OR EPA [Title/Abstract] OR Docosahexaenoic [Title/Abstract] OR DHA [Title/Abstract] OR Flavonol* [Title/Abstract] OR Lignan* [Title/Abstract] OR Oleic [Title/Abstract] OR soy* [Title/Abstract] OR catechin* [Title/Abstract] OR DASH diet* [Title/Abstract] OR plant sterol* [Title/Abstract] OR grape* [Title/Abstract] OR Probiotic [Title/Abstract] OR Pistachio [Title/Abstract] OR Resveratrol [Title/Abstract] OR Milk [Title/Abstract] OR Beef [Title/Abstract] OR Fiber* [Title/Abstract] OR inulin [Title/Abstract] OR margarine* [Title/Abstract] OR Garlic [Title/Abstract]) AND humans [MeSH Terms]

8. HDL-associated phospholipase A2

(high density [Title/Abstract] OR high-density [Title/Abstract] OR HDL* [Title/Abstract]) AND (platelet-activating factor acetyl*[Title/Abstract] OR *PAFAH* [Title/Abstract] OR *PAF-AH* [Title/Abstract] OR *LpPLA2* [Title/Abstract] OR *Lp-PLA2* [Title/Abstract] OR HDL-Lp-PLA2 [Title/Abstract] OR lipoprotein-associated phospholipase [Title/Abstract]) AND (diet* [Title/Abstract] OR nutr* [Title/Abstract] OR fatty acid* [Title/Abstract] OR oil [Title/Abstract] OR *saturated [Title/Abstract] OR trans [Title/Abstract] OR MUFA* [Title/Abstract] OR PUFA* [Title/Abstract] OR omega* [Title/Abstract] OR antioxidant [Title/Abstract] OR vitamin [Title/Abstract] OR tocopher*

[Title/Abstract] OR ascorb* [Title/Abstract] OR lycopene [Title/Abstract] OR *phenol [Title/Abstract] OR *phenols [Title/Abstract] OR phenol* [Title/Abstract] OR flavonoid* [Title/Abstract] OR fruit* [Title/Abstract] OR vegeta* [Title/Abstract] OR juice* [Title/Abstract] OR *berry [Title/Abstract] OR olive [Title/Abstract] OR tomato [Title/Abstract] OR tea [Title/Abstract] OR coffee [Title/Abstract] OR whole grain* [Title/Abstract] OR legume* [Title/Abstract] OR fish [Title/Abstract] OR egg* [Title/Abstract] OR nut [Title/Abstract] OR walnut* [Title/Abstract] OR *nuts [Title/Abstract] OR low fat [Title/Abstract] OR protein-rich [Title/Abstract] OR Mediterranean diet [Title/Abstract] OR ethanol* [Title/Abstract] OR alcohol* [Title/Abstract] OR beer [Title/Abstract] OR wine [Title/Abstract] OR lifestyle [Title/Abstract] OR physical activity [Title/Abstract] OR exercise [Title/Abstract] OR training [Title/Abstract] OR weight loss [Title/Abstract] OR restrict* [Title/Abstract] OR very low calorie [Title/Abstract] OR Eicosapentaenoic [Title/Abstract] OR EPA [Title/Abstract] OR Docosahexaenoic [Title/Abstract] OR DHA [Title/Abstract] OR Flavonol* [Title/Abstract] OR Lignan* [Title/Abstract] OR Oleic [Title/Abstract] OR soy* [Title/Abstract] OR catechin* [Title/Abstract] OR DASH diet* [Title/Abstract] OR plant sterol* [Title/Abstract] OR grape* [Title/Abstract] OR Probiotic [Title/Abstract] OR Pistachio [Title/Abstract] OR Resveratrol [Title/Abstract] OR Milk [Title/Abstract] OR Beef [Title/Abstract] OR Fiber* [Title/Abstract] OR inulin [Title/Abstract] OR margarine* [Title/Abstract] OR Garlic [Title/Abstract]) AND humans [MeSH Terms]

9. HDL-associated with SAA

(high density [Title] OR high-density [Title] OR HDL* [Title]) AND (amyloid [Title/Abstract] OR SAA* [Title/Abstract] OR *SAA [Title/Abstract]) AND (diet* [Title/Abstract] OR nutr* [Title/Abstract] OR fatty acid* [Title/Abstract] OR oil [Title/Abstract] OR *saturated [Title/Abstract] OR trans [Title/Abstract] OR MUFA* [Title/Abstract] OR PUFA* [Title/Abstract] OR omega* [Title/Abstract] OR antioxidant [Title/Abstract] OR vitamin [Title/Abstract] OR tocopher* [Title/Abstract] OR ascorb* [Title/Abstract] OR lycopene [Title/Abstract] OR *phenol [Title/Abstract] OR *phenols [Title/Abstract] OR phenol* [Title/Abstract] OR flavonoid* [Title/Abstract] OR fruit*

[Title/Abstract] OR vegeta* [Title/Abstract] OR juice* [Title/Abstract] OR *berry [Title/Abstract] OR olive [Title/Abstract] OR tomato [Title/Abstract] OR tea [Title/Abstract] OR coffee [Title/Abstract] OR whole grain* [Title/Abstract] OR legume* [Title/Abstract] OR fish [Title/Abstract] OR egg* [Title/Abstract] OR nut [Title/Abstract] OR walnut* [Title/Abstract] OR *nuts [Title/Abstract] OR low fat [Title/Abstract] OR protein-rich [Title/Abstract] OR Mediterranean diet [Title/Abstract] OR ethanol* [Title/Abstract] OR alcohol* [Title/Abstract] OR beer [Title/Abstract] OR wine [Title/Abstract] OR lifestyle [Title/Abstract] OR physical activity [Title/Abstract] OR exercise [Title/Abstract] OR training [Title/Abstract] OR weight loss [Title/Abstract] OR restrict* [Title/Abstract] OR very low calorie [Title/Abstract] OR Eicosapentaenoic [Title/Abstract] OR EPA [Title/Abstract] OR Docosahexaenoic [Title/Abstract] OR DHA [Title/Abstract] OR Flavonol* [Title/Abstract] OR Lignan* [Title/Abstract] OR Oleic [Title/Abstract] OR soy* [Title/Abstract] OR catechin* [Title/Abstract] OR DASH diet* [Title/Abstract] OR plant sterol* [Title/Abstract] OR grape* [Title/Abstract] OR Probiotic [Title/Abstract] OR Pistachio [Title/Abstract] OR Resveratrol [Title/Abstract] OR Milk [Title/Abstract] OR Beef [Title/Abstract] OR Fiber* [Title/Abstract] OR inulin [Title/Abstract] OR margarine* [Title/Abstract] OR Garlic [Title/Abstract]) AND humans [MeSH Terms]

10 HDL sphingosine-1-phosphate content

(high density [Title] OR high-density [Title] OR HDL* [Title]) AND (sphingosin* [Title/Abstract] OR S1P* [Title/Abstract] OR *S1P [Title/Abstract]) AND (diet* [Title/Abstract] OR nutr* [Title/Abstract] OR fatty acid* [Title/Abstract] OR oil [Title/Abstract] OR *saturated [Title/Abstract] OR trans [Title/Abstract] OR MUFA* [Title/Abstract] OR PUFA* [Title/Abstract] OR omega* [Title/Abstract] OR antioxidant [Title/Abstract] OR vitamin [Title/Abstract] OR tocopher* [Title/Abstract] OR ascorb* [Title/Abstract] OR lycopene [Title/Abstract] OR *phenol [Title/Abstract] OR *phenols [Title/Abstract] OR phenol* [Title/Abstract] OR flavonoid* [Title/Abstract] OR fruit*

[Title/Abstract] OR vegeta* [Title/Abstract] OR juice* [Title/Abstract] OR *berry [Title/Abstract] OR olive [Title/Abstract] OR tomato [Title/Abstract] OR tea [Title/Abstract] OR coffee [Title/Abstract] OR whole grain* [Title/Abstract] OR legume* [Title/Abstract] OR fish [Title/Abstract] OR egg* [Title/Abstract] OR nut [Title/Abstract] OR walnut* [Title/Abstract] OR *nuts [Title/Abstract] OR low fat [Title/Abstract] OR protein-rich [Title/Abstract] OR Mediterranean diet [Title/Abstract] OR ethanol* [Title/Abstract] OR alcohol* [Title/Abstract] OR beer [Title/Abstract] OR wine [Title/Abstract] OR lifestyle [Title/Abstract] OR physical activity [Title/Abstract] OR exercise [Title/Abstract] OR training [Title/Abstract] OR weight loss [Title/Abstract] OR restrict* [Title/Abstract] OR very low calorie [Title/Abstract] OR Eicosapentaenoic [Title/Abstract] OR EPA [Title/Abstract] OR Docosahexaenoic [Title/Abstract] OR DHA [Title/Abstract] OR Flavonol* [Title/Abstract] OR Lignan* [Title/Abstract] OR Oleic [Title/Abstract] OR soy* [Title/Abstract] OR catechin* [Title/Abstract] OR DASH diet* [Title/Abstract] OR plant sterol* [Title/Abstract] OR grape* [Title/Abstract] OR Probiotic [Title/Abstract] OR Pistachio [Title/Abstract] OR Resveratrol [Title/Abstract] OR Milk [Title/Abstract] OR Beef [Title/Abstract] OR Fiber* [Title/Abstract] OR inulin [Title/Abstract] OR margarine* [Title/Abstract] OR Garlic [Title/Abstract]) AND humans [MeSH Terms]

11. HDL-alpha-1-antitrypsin

(high density [Title] OR high-density [Title] OR HDL* [Title]) AND (*trypsin [Title/Abstract]) AND (diet* [Title/Abstract] OR nutr* [Title/Abstract] OR fatty acid* [Title/Abstract] OR oil [Title/Abstract] OR *saturated [Title/Abstract] OR trans [Title/Abstract] OR MUFA* [Title/Abstract] OR PUFA* [Title/Abstract] OR omega* [Title/Abstract] OR antioxidant [Title/Abstract] OR vitamin [Title/Abstract] OR tocopher* [Title/Abstract] OR ascorb* [Title/Abstract] OR lycopene [Title/Abstract] OR *phenol [Title/Abstract] OR *phenols [Title/Abstract] OR phenol* [Title/Abstract] OR flavonoid* [Title/Abstract] OR fruit* [Title/Abstract] OR vegeta* [Title/Abstract] OR juice*

[Title/Abstract] OR *berry [Title/Abstract] OR olive [Title/Abstract] OR tomato [Title/Abstract] OR tea [Title/Abstract] OR coffee [Title/Abstract] OR whole grain* [Title/Abstract] OR legume* [Title/Abstract] OR fish [Title/Abstract] OR egg* [Title/Abstract] OR nut [Title/Abstract] OR walnut* [Title/Abstract] OR *nuts [Title/Abstract] OR low fat [Title/Abstract] OR protein-rich [Title/Abstract] OR Mediterranean diet [Title/Abstract] OR ethanol* [Title/Abstract] OR alcohol* [Title/Abstract] OR beer [Title/Abstract] OR wine [Title/Abstract] OR lifestyle [Title/Abstract] OR physical activity [Title/Abstract] OR exercise [Title/Abstract] OR training [Title/Abstract] OR weight loss [Title/Abstract] OR restrict* [Title/Abstract] OR very low calorie [Title/Abstract] OR Eicosapentaenoic [Title/Abstract] OR EPA [Title/Abstract] OR Docosahexaenoic [Title/Abstract] OR DHA [Title/Abstract] OR Flavonol* [Title/Abstract] OR Lignan* [Title/Abstract] OR Oleic [Title/Abstract] OR soy* [Title/Abstract] OR catechin* [Title/Abstract] OR DASH diet* [Title/Abstract] OR plant sterol* [Title/Abstract] OR grape* [Title/Abstract] OR Probiotic [Title/Abstract] OR Pistachio [Title/Abstract] OR Resveratrol [Title/Abstract] OR Milk [Title/Abstract] OR Beef [Title/Abstract] OR Fiber* [Title/Abstract] OR inulin [Title/Abstract] OR margarine* [Title/Abstract] OR Garlic [Title/Abstract]) AND humans [MeSH Terms]

12. HDL-associated complement proteins

(high density [Title] OR high-density [Title] OR HDL* [Title]) AND (complement [Title/Abstract]) AND (diet* [Title/Abstract] OR nutr* [Title/Abstract] OR fatty acid* [Title/Abstract] OR oil [Title/Abstract] OR *saturated [Title/Abstract] OR trans [Title/Abstract] OR MUFA* [Title/Abstract] OR PUFA* [Title/Abstract] OR omega* [Title/Abstract] OR antioxidant [Title/Abstract] OR vitamin [Title/Abstract] OR tocopher* [Title/Abstract] OR ascorb* [Title/Abstract] OR lycopene [Title/Abstract] OR *phenol [Title/Abstract] OR *phenols [Title/Abstract] OR phenol* [Title/Abstract] OR flavonoid* [Title/Abstract] OR fruit* [Title/Abstract] OR vegeta* [Title/Abstract] OR juice* [Title/Abstract] OR *berry [Title/Abstract] OR olive [Title/Abstract] OR tomato

[Title/Abstract] OR tea [Title/Abstract] OR coffee [Title/Abstract] OR whole grain* [Title/Abstract] OR legume* [Title/Abstract] OR fish [Title/Abstract] OR egg* [Title/Abstract] OR nut [Title/Abstract] OR walnut* [Title/Abstract] OR *nuts [Title/Abstract] OR low fat [Title/Abstract] OR protein-rich [Title/Abstract] OR Mediterranean diet [Title/Abstract] OR ethanol* [Title/Abstract] OR alcohol* [Title/Abstract] OR beer [Title/Abstract] OR wine [Title/Abstract] OR lifestyle [Title/Abstract] OR physical activity [Title/Abstract] OR exercise [Title/Abstract] OR training [Title/Abstract] OR weight loss [Title/Abstract] OR restrict* [Title/Abstract] OR very low calorie [Title/Abstract] OR Eicosapentaenoic [Title/Abstract] OR EPA [Title/Abstract] OR Docosahexaenoic [Title/Abstract] OR DHA [Title/Abstract] OR Flavonol* [Title/Abstract] OR Lignan* [Title/Abstract] OR Oleic [Title/Abstract] OR soy* [Title/Abstract] OR catechin* [Title/Abstract] OR DASH diet* [Title/Abstract] OR plant sterol* [Title/Abstract] OR grape* [Title/Abstract] OR Probiotic [Title/Abstract] OR Pistachio [Title/Abstract] OR Resveratrol [Title/Abstract] OR Milk [Title/Abstract] OR Beef [Title/Abstract] OR Fiber* [Title/Abstract] OR inulin [Title/Abstract] OR margarine* [Title/Abstract] OR Garlic [Title/Abstract]) AND humans [MeSH Terms]
